# Infection patterns of scabies and tinea between inland and resettled indigenous Negrito communities in Peninsular Malaysia

**DOI:** 10.1371/journal.pntd.0012515

**Published:** 2024-09-26

**Authors:** Yi Xian Er, Leslie Thian Lung Than, Azdayanti Muslim, Nan Jiun Yap, Mian Zi Tee, Nurmanisha Abdull-Majid, Soo Ching Lee, Shezryna Shahrizal, Yvonne Ai Lian Lim

**Affiliations:** 1 Department of Parasitology, Faculty of Medicine, Universiti Malaya, Kuala Lumpur, Malaysia; 2 Department of Medical Microbiology, Universiti Putra Malaysia, UPM Serdang, Selangor Darul Ehsan, Malaysia; 3 Department of Medical Microbiology and Parasitology, Faculty of Medicine, Universiti Teknologi MARA (Sungai Buloh Campus), Sungai Buloh, Malaysia; 4 Institute for Biodiversity and Sustainable Development, Universiti Teknologi MARA, Shah Alam, Selangor, Malaysia; 5 Type 2 Immunity Section, Laboratory of Parasitic Diseases, National Institute of Allergy and Infectious Diseases, National Institute of Health, Bethesda, Maryland, United States of America; 6 Centre for Malaysian Indigenous Studies, Universiti Malaya, Kuala Lumpur, Malaysia; Albert Einstein College of Medicine, UNITED STATES OF AMERICA

## Abstract

Skin infections cause significant health burden and affect underserved communities such as the indigenous Negrito communities disproportionately. There is only one study that has addressed skin infections among the Negrito communities, which is the smallest and most isolated indigenous tribe in Peninsular Malaysia, with approximately 6,500 individuals remaining in northern and central Peninsular Malaysia. This study, which aims to update the infection patterns of scabies and tinea among the Negrito communities, recruited 361 participants from eight villages representing all six Negrito subtribes. The results revealed an overall skin infection prevalence of 35.6%, with scabies (11.7%), tinea versicolor (11.3%), and tinea imbricata (7.5%) as major infections, with no co-infection. Notably, infection rates were significantly higher in resettled villages (55.2%) compared to inland villages (24.8%). Scabies and tinea versicolor were more prevalent in resettled villages (21.2% and 23.6%, respectively) than inland villages (6.4% and 4.7%, respectively), while tinea imbricata was more common in inland villages (9.4% vs 3.9%). Furthermore, there exist predisposition of scabies among Kensiu. High prevalence of tinea imbricata was observed among the inland Bateq while prevalence of tinea versicolor was high among the resettled Bateq. Risk analysis revealed specific associations: scabies with Kensiu subtribe (P = 0.002), high income (P = 0.001) and underweight individuals (P = 0.009); tinea versicolor with Bateq subtribe (P = 0.003), resettled villagers (P < 0.001), males (P = 0.040), and overweight/obese individuals (P = 0.015); and tinea imbricata with Bateq (P = 0.011) and smokers (P = 0.004). These findings highlight a complex interplay between environment and lifestyle in skin infection prevalence. Addressing these infections requires targeted interventions, including regular medical care in inland villages and socio-economic support for resettled communities, considering the distinct predispositions in different village types.

## Author summary

Skin infections (tinea and scabies) are significant health concerns among the indigenous Negrito communities in Peninsular Malaysia. Given that there was only one documented study among Negrito communities previously, the present study enrolled 361 participants from eight Negrito categorised into resettled or inland villages in Peninsular Malaysia to update the epidemiological status and risk factors. We found a high overall skin infection prevalence of 35.6%. Scabies, tinea versicolor, and tinea imbricata accounted for 11.7%, 11.3%, and 7.5%, respectively with no co-infection. Higher prevalence was observed among resettled villages (55.2%) compared to inland villages (24.8%). Risk factor analysis among these Negrito communities identified different predictors associated with each type of infection, generally linked to the type of village category. We highlighted that scabies and tinea versicolor were more common in resettled communities. However, rare infection like tinea imbricata, which require high treatment compliance, was more localized in inland villages. Village-specific measures including regular medical interventions in inland villages and socio-economic aid to resettled communities to address urban/semi-urban poverty are essential, considering the existing predisposition of skin infections in different types of villages.

## Introduction

Skin infections refer to various cutaneous infection which include scabies, tinea and impetigo. These skin infections have been associated with draw-back in socio-economic development and infrastructure availability [1]. Hence, the indigenous populations of both the developed and developing countries are often the group who are deeply affected by these skin infections. For example, the prevalence of scabies, a common skin infections caused by *Sarcoptes scabiei* can be as high as 33.0% among the Australian indigenous communities [2] whereas tinea imbricata, a rare infections which is almost never found among the general population, has a prevalence as high as 61.0% among Dayak tribe in Indonesia [3], 18.7% among Negrito tribe in Malaysia [4], and up to 20.0% among the indigenous people in Papua New Guinea [5].

Authorities often underestimate the burden of skin infections due to their relatively low mortality rates [6]. However, skin infections like scabies and tinea may have significant impacts on the affected communities in terms of health, psychology, and the economy. Some of the most common skin infections among the Orang Asli is tinea [7], which is a group of skin fungal infection caused by dermatophyte such as *Trichophyton* and *Microsporum* or yeast like *Malasezzia* [8]. Colonization of the dermatophyte often lead to shedding of host epidermal layer and itchiness [9]. The clinical manifestation of the lesion also varies according to site, which form the basis of the disease name, include tinea capitis (body site: head, signs: balding of scalp), tinea unguium (body site: finger, signs: grey-coloration and corrosion of the fingernail) and tinea pedis (body site: feet area, signs: rash and desquamation of the area). In the case of tinea infections, the immune system’s response to the fungus could trigger a secondary skin reaction called dermatophytid, characterize by erythematous rashes [10]. Another notable skin infection among these underserved communities is scabies, which is a skin infection caused by human mites known as *Sarcoptes scabiei* [11]. These mites burrow and reside within the host’s epidermis layer, feeding on host secretions including sebum, leading to pruritus and inflammation [12], and hence results in the formation of papules vesicles in the affected areas and eczema [13]. Often the patients would scratch the affected areas, causing the formation of impetigo in the affected areas [14]. Skin infections are often associated with negative images of inadequate level of cleanliness and poverty, leading to isolation and stigmatization by communities, which could have long term psychological effects on patients even after recovery [15].

Given the preponderance of skin infections amongst the indigenous communities, this study attempted to elucidate the status of skin infections among the underserved indigenous, namely the Orang Asli. The Orang Asli is a group of indigenous people living in Peninsular Malaysia with an approximate population size of 215,215 [16]. There are 3 main tribes, namely Senoi, Proto-Malay and Negrito and within each main tribe, there are six subtribes as shown in S1 Table. Similar to other indigenous population around the globe, Orang Asli suffers from a variety of skin infections which include dermatomycoses [7] and scabies [17] due to their traditional practices such as barefooting and pets/animal husbandry techniques, high level of poverty (76.9% of them are experiencing poverty) [18] and backwardness in development [19].

Skin infections among the Orang Asli were first reported in 1952 by Sharvil et al. [20]. In the same year, an epidemiological study was conducted by Polunin among the tribes, including the Lanoh subtribe (Negrito tribe), Semai subtribe (Senoi tribe), and Orang Seletar subtribe (Proto-Malay tribe). The prevalence of tinea was approximately 17.4% among 581 villagers from four villages, with tinea imbricata (9.1%) being the most common, followed by ringworm (2.8%) and tinea versicolor (5.5%). The distribution of tinea varied according to tribes and villages [4]. The study also highlighted that the prevalence of the skin infections among the Negrito communities were the highest (18.3%), followed by Semai (1.7–9.9%) and Proto-Malay (4.4%). In 1998, a study among 356 participants from the Proto-Malay tribe at Sungai Siput, Perak highlighted that 3.5% of the villagers had ringworm, 1.5% of them had tinea versicolor, 7% of them with impetigo and 11.9% of the villagers having scabies [17]. Both these studies used microscopy and medical observation to diagnose the skin infections. There was also a sero-prevalence survey of the scabies among the indigenous communities in 1996, which showed that 24.7% of the 312 participants were seropositive for anti-scabies antibodies [21].

The Negrito Orang Asli, the focus of our study, is the smallest tribe with an approximate total population of 6,500 (3.0% of the entire Orang Asli population) [16]. Of the six subtribes, the second largest subtribe of Negritos, Bateq (n = 2,190) are still practicing semi-nomadic lifestyles with some communities living in remote inland forest areas [22], whereas other subtribes, namely Jahai (n = 2,915) and Mandriq (n = 531) are inland agrarian. A previous study conducted in 1952 found that the prevalence of skin infections was highest among the Negrito Orang Asli tribe in Malaysia, with 18.7% of the population affected [4]. Tinea imbricata was the most common infection (18.3%), followed by tinea versicolor (6.1%) and tinea circinata (2.4%). As this data has not been updated in the past 72 years for the Negrito tribe, the present study is carried out amongst the Negrito Orang Asli tribe. With limited epidemiology studies conducted among the Orang Asli and only one study carried out among the Negrito tribe, there is a crucial need to update and fill the gap of knowledge and information regarding the current status of skin infections in these indigenous communities. Secondly, media news coverage and case reports over the past 5 years have highlighted skin infections among the Negrito communities [23–26] with no up-to-date thorough studies as evidence. Furthermore, prior study did not analyse the risk factors which were influencing these infections. Current available associated factors analysis with regards to tinea and scabies are either conducted among general population only.

Henceforth, a cross-sectional study was conducted to investigate the current status and risk factors of skin infections among indigenous Negrito communities in Peninsular Malaysia. The Negrito villages included in this study ranged from those in the inland and those in the resettled villages. They also comprised all the 6 subtribes within the Negrito tribe, namely Mandriq, Bateq, Jahai, Kensiu, Kintaq, Lanoh to increase the coverage of representation of the Negrito communities. The findings of this study provided updated data on skin infections among indigenous people, which would be beneficial for public health officials in developing effective targeted and customized control programs to reduce skin infections among these underserved communities.

## Results

### Demographic data of the participants and study areas

As illustrated in Table 1, a total of 361 participants were recruited from 8 villages and each sampling site shows varying level of development status, income and main economic activity. There was a total of 5 inland villages (Villages A-E, with a total of 234 participants) and 3 resettled villages (Villages F-H, with a total of 127 participants). A majority (52.6%) of the participants were of Bateq subtribe, comprising of Bateq people in both resettled villages (Village G) and inland villages (Villages A-D). Kensiu, Kintak and Jahai subtribes were only found in Villages F, E and H, respectively. Participants from Mandriq and Lanoh subtribes were found in Village A, F and G.

**Table 1:** Basic demographic data of each Orang Asli village under the categories of inland and resettled villages.

**Village Status**		**Inland Villages**					**Resettled Villages**			**Total (N = 361)**
**Village**		**A**	**B**	**C**	**D**	**E**	**F**	**G**	**H**	
		(N = 40)	(N = 66)	(N = 38)	(N = 12)	(N = 78)	(N = 40)	(N = 51)	(N = 36)	
		n (%)	n (%)	n (%)	n (%)	n (%)	n (%)	n (%)	n (%)	N (%)
**Gender**	Female	17 (42.5)	29 (43.9)	23 (60.5)	5 (41.7)	45 (57.7)	20 (50.0)	6 (11.8)	19 (52.8)	164 (45.4)
	Male	23 (57.5)	37 (56.1)	15 (39.5)	7 (58.3)	33(42.3)	20 (50.0)	45 (88.2)	17 (47.2)	197 (54.6)
**Subtribe**	Bateq	32 (80.0)	66 (100.0)	38 (100.0)	12 (100.0)	0	0	42 (82.4)	0	190 (52.6)
	Jahai	0	0	0	0	0	0	0	36 (100.0)	36 (10.0)
	Kensiu	0	0	0	0	0	35 (87.5)	0	0	35 (9.7)
	Kintak	0	0	0	0	78 (100.0)	0	0	0	78 (21.6)
	Mandriq & Lanoh	8 (20.0)	0	0	0	0	5 (12.5)	9 (17.6)	0	22 (6.1)
							
**Age**	Adult	19 (47.5)	23 (34.8)	25 (65.8)	12 (100.0)	39 (50.0)	29 (72.5)	39 (76.5)	24 (66.7)	210 (58.2)
**Group**	Kids and teenagers	21 (52.5)	43 (65.2)	13 (34.2)	0	39 (50.0)	11 (27.5)	12 (23.5)	12 (33.3)	151 (41.8)
**Body Mass Index**	Normal	4 (10.0)	14 (21.2)	7 (18.4)	8 (66.7)	33 (42.3)	11 (27.5)	9 (17.6)	14 (38.9)	100 (27.7)
	Underweight	22 (55.0)	52 (78.8)	31 (81.6)	3 (25.0)	27 (34.6)	16 (40.0)	15 (29.4)	12 (33.3)	178 (49.3)
	Obese & overweight	14 (35.0)	0	0	1 (8.3)	18 (23.1)	13 (32.5)	27 (52.9)	10 (27.8)	83 (23.0)
**Occupation**	Agriculture	11 (27.5)	1 (1.5)	0	11 (91.7)	16 (20.5)	9 (22.5)	21 (41.2)	11 (30.6)	80 (22.2)
	Forestry	4 (10.0)	20 (30.3)	13 (34.2)	1 (8.3)	5 (6.4)	3 (7.5)	0	3 (8.3)	49 (13.6)
	Housewives	7 (17.5)	4 (6.1)	13 (34.2)	0	17 (21.8)	8 (20.0)	6 (11.8)	9 (25.0)	64 (17.7)
	Others	0	0	2 (5.3)	0	2 (206)	9 (22.5)	6 (11.8)	0	19 (5.3)
	Retiree	0	0	0	0	0	0	6 (11.8)	0	6 (1.7)
	Student	18 (45.0)	41 (62.1)	10 (26.3)	0	38 (48.7)	11 (27.5)	12 (23.5)	13 (36.1)	143 (39.6)
**Water Usage**	Treated	0	0	0	9(75.0)	0	6 (15.0)	24 (47.1)	0	42 (11.6)
	Untreated	40(100.0)	66(100.0)	12 (31.6)	0	78(100.0)	8 (20.0)	0	36 (100.0)	240 (66.5)
	Mix	0	0	26 (68.4)	3 (25.0)	0	26(65.0)	27 (52.9)	0	79(21.8%)
**Income**	<MYR 800	38 (95.0)	66 (100.0)	31 (81.6)	12 (100.0)	78 (100.0)	25 (62.5)	39 (76.5)	36 (100.0)	325 (90.0)
	> MYR 800	2 (5.0)	0	7 (18.4)	0	0	15 (37.5)	12 (23.5)	0	36 (10.0)
**Education**	No formal education	16 (40.0)	37 (56.1)	22 (57.9)	12 (100.0)	43 (55.1)	13 (32.5)	15 (29.4)	5 (13.9)	163 (45.2)
	With education	24 (60.0)	29 (43.9)	16 (42.1)	0	35 (44.9)	27 (67.5)	36 (70.6)	31 (86.1)	198 (54.8)

*Adult refer to group that are ≥ 20 years old whereas teen and kids were the group < 20 years old

* Inland villages in general refer to the villages that were not part of the resettled program and are situated in their original locations, whereas resettled villages refer to the villages that participated in the relocation program.

Among the participants, 54.6% were male and 45.4% were female. The majority of the participants were either adults (>21 years old, 58.2%) or children (<9 years old, 41.8%). Slightly more than half of them (59.2%) were not working, with most being students, housewives, and retirees. An overwhelmingly majority of the participants (90.0%) belonged to households with a monthly household income of less than MYR 800, which is below the poverty income threshold (MYR 1,169) in Malaysia [27–29], a possible reason for the high number of underweight participants. In terms of educational attainment, 54.8% had formal education, while 45.2% of them never attended school.

The data also revealed that 21.8% of the study participants were using both treated and untreated water, while only 11.6% relied solely on treated water. This pattern was particularly prevalent in the more urbanized villages of Village G and Village F, where 52.9% and 65.0% of the participants, respectively, reported using both treated and untreated water in their daily lives due to urban poverty (water supply cut due to inability to pay utility bills). This information highlighted the irony that it was not necessarily inland villages that solely relied on untreated water and resettled villages relied on treated water as there were inland villages such as Village C and Village D using both treated (water from nearby tourism chalet) and untreated water and resettled village such as Village F which relied on untreated water (with support of modern water delivery system).

### Overall prevalence of scabies and tinea

Overall, 35.6% (CI = 30.5% - 40.6%) of the 361 participants had skin infections. The most common skin infections were scabies (11.6%; 95% CI = 8.6% - 15.5%), tinea versicolor (11.4%; 95% CI = 8.4% - 15.2%) and tinea imbricata (7.5%, 95% CI = 5.0% - 10.8%), as shown in Table 2. When the analysis was further stratified, it was found that the prevalence of skin infections in resettled villages was higher (55.2%) as compared to inland villages (24.8%). Specifically, both scabies and tinea versicolor were more pronounced (21.2% and 23.6%, respectively) in the resettled Negrito villages versus inland villages (6.4% and 4.7%, respectively) (P < 0.001). In contrast, tinea imbricata was found to be higher in the inland (9.4%) versus resettled villages (3.9%) (P = 0.049). It was interesting to note that there was no co-infection of tinea and scabies among the Negrito Orang Asli.

**Table 2:** Prevalence and 95% confidence interval of skin infections among the participants, in accordance with the different villages. Scabies and tinea versicolor were more prevalent among the resettled villages, whereas tinea imbricata was mostly found in inland villages.

				Overall skin infections n (%)		Type of Skin Infection n (%)			
Variables		Description	Total Number/ N	Positive	Negative	Scabies	Tinea versicolor	Tinea imbricata	Other Tinea & skin diseases
**Village**	**Inland Villages**	**A**	40	8 (20.00)	32 (80.00)	3 (7.50)	4 (10.00)	0	1 (2.50)
		**B**	66	6 (9.09)	60 (90.91)	2 (3.03)	2 (3.03)	2 (3.03)	0
		**C**	38	28 (73.68)	10 (26.32)	3 (7.89)	0	16 (42.11)	9 (23.68)
		**D**	12	10 (83.33)	2 (16.67)	0 (0.00)	5 (41.67)	4 (33.33)	1 (8.33)
		**E**	78	6 (7.89)	71 (92.11)	7 (8.97)	0	0	0
	**Resettled Villages**	**F**	40	34 (85.00)	6 (15.00)	24 (60.00)	0	5 (12.50)	5 (12.50)
		**G**	51	30 (58.82)	21 (41.18)	3 (5.88)	24 (47.06)	0	3 (8.33)
		**H**	36	6 (16.67)	30 (92.11)	0	6 (16.67)	0	0
**Subtribe**		**Bateq**	190	73 (38.42)	117 (61.58)	8 (4.21)	30 (15.79)	22 (11.58)	13 (6.84)
		**Jahai**	36	6 (16.67)	30 (83.33)	0	6 (16.67)	0	0
		**Kensiu**	35	29 (82.86)	6 (17.14)	19 (54.29)	0	5 (14.29)	5 (14.29)
		**Kintak**	78	7 (8.97)	71 (91.03)	7 (8.97)	0	0	0
		**Mandriq & Lanoh**	22	14 (63.64)	8 (36.36)	8 (36.36)	5 (22.72)	0	1 (4.55)
**Age group**		**Adult**	210	85 (40.48)	125 (59.52)	22 (10.48)	28 (13.33)	19 (9.05)	16 (7.62)
		**Kids and teenagers**	151	44 (29.14)	107 (70.86)	20 (13.24)	13 (8.61)	8 (5.30)	3 (1.99)
**Gender**		**Male**	197	80 (40.61)	117 (59.39)	23 (11.68)	32 (16.24)	12 (6.09)	13 (6.60)
		**Female**	164	49 (29.89)	115 (70.11)	19 (11.56)	9 (5.49)	15 (9.15)	6 (3.66)
Total			361	128 (35.46)	233 (64.54)	42 (11.63)	41 (11.36)	27 (7.48)	19 (5.26)
95% CI (Based on total)				30.57–40.66	59.33–69.43	8.60–15.51	8.36–15.20	5.08–10.82	3.29–8.24

*Tinea and others: tinea refer to those who are positive in potassium hydroxide test but negative in CLSI-54a test whereas others refer to those with symptoms but negative in both tests

* Inland villages in general refer to the villages that were not part of the resettled program and are situated in their original locations, whereas resettled villages refer to the villages that participated in the relocation program.

As illustrated in Table 2, huge variations in terms of scabies and tinea distributions were demonstrated among the villages. More than 70% of the participants in villages C, D and F had scabies and tinea, whereas village A had the lowest prevalence (7.7%). The Kensiu subtribe appeared to have the highest prevalence (60.0%) of skin infections. This Kensiu subtribe community resides in Village F, a resettled village with basic amenities such as treated water and electricity, as well as walking distance to health and education (primary and secondary) facilities.

It was also observed that Bateq subtribe living in different villages displayed predisposition toward different skin infections. For example, those Bateq living in resettled Village G displayed high prevalence of tinea versicolor (47.06%) whereas Bateq living in inland Villages C (42.11%) and D (33.33%) displayed high prevalence of tinea imbricata. There was a higher prevalence of scabies and tinea versicolor among the obese and overweight group (prevalence of 13.3% in scabies and 24.1% in tinea versicolor) and underweight participants (prevalence of 14.6% in scabies and 9.0% in tinea versicolor) than those with normal BMI (prevalence of 5.0% in both scabies and tinea versicolor). `

### Prevalence and risk factor analysis of scabies, tinea versicolor and tinea imbricata

#### a Scabies

In this study, scabies accounted for 11.6% of the prevalence rate, with 60.0% of cases belonging to the Kensiu subtribe who reside in the more urbanized village, Village F. On the other hand, its prevalence was fairly consistent across all other villages, remaining at around 3–7%. Interestingly, Villages D (Inland) and H (resettled) were free from scabies infections. There were 54.3% of the Kensiu participants and 36.4% of the Mendriq/Lanoh participants who had scabies, while the Bateq participants had the lowest prevalence of scabies (4.2%).

In the univariate analysis (Table 3), we found that individuals from the Kensiu subtribe (COR = 18.9, P < 0.001), underweight (COR = 3.3, P = 0.02), an income level less than RM 800 (COR = 7.9, P < 0.001), and those living in resettled villages had higher odds of scabies infections compared to their counterparts (COR = 3.9, P < 0.001). However, in the multivariate analysis, only three factors remained significant predictors of scabies infections: Kensiu subtribe (AOR = 7.7, P = 0.002), underweight (AOR = 9.7, P = 0.009) and income of more than RM 800 (AOR = 7.5, P = 0.001).

**Table 3:** Potential risk factors associated with scabies based on logistic regression analysis.

Variable	N	Scabies infected, n (%)	Univariate		Multivariate	
			COR (CI)	P value	AOR (CI)	P value
**Kintak subtribe**					Nil	
No	283	35 (12.4)	1	0.41		
Yes	78	7 (9.0)	0.7 (0.3–1.6)			
**Kensiu subtribe**						
No	324	21 (6.5)	1		1	
Yes	37	21 (56.8)	18.9 (8.7–42.4)	<0.001*	7.7(2.3–29.3)	0.002*
**Bateq subtribe**						
No	168	31 (18.5)	1		1	0.07
Yes	193	11 (5.7)	0.3 (0.1–0.5)	<0.001*	0.4(0.1–1.0)	
**Gender**					Nil	
female	164	19 (11.6)	1	0.979		
male	197	23 (11.7)	1.0 (0.5–1.9)			
**Body Mass Index**						
Normal	100	5 (5.0)	1		1	
Obese & overweight	83	11 (13.3)	2.9 (1.0–9.5)	0.058	3.2 (0.8–14.1)	0.109
Underweight	178	26 (14.6)	3.3 (1.3–9.9)	0.020*	9.7 (3.0–41.1)	0.009*
**Age group**					Nil	
Adult	210	22 (10.5)	1	0.419		
Kids and teens	151	20 (13.2)	1.3 (0.7–2.5)			
**Income level**						
<RM800	325	27 (8.3)	1	<0.001*	1	
>RM800	36	15 (41.7)	7.9 (3.6–17.1)		7.5(2.3–25.0)	0.001*
**Presence of pets**					Nil	
No	144	15 (10.4)	1	0.557		
Yes	217	27 (12.4)	1.2 (0.6–2.4)			
**Presence of family member with the same disease**					Nil	
No	200	26 (13.0)	1	0.368		
Yes	161	16 (9.9)	0.7 (0.4–1.4)			
**Usage of topical ointments**					Nil	
No	244	18 (7.4)	1	<0.001*		
Yes	117	24 (20.5)	3.2 (1.7–6.3)			
**Village status**						
Inland village	234	15 (6.4)	1	<0.001*	1	0.896
Resettled village	127	27 (21.3)	3.9 (2.0–7.9)		1.1 (0.3–3.2)	
**Occupations**					Nil	
Away from village	148	12 (8.1)	1	0.085		
Within village	213	30 (14.1)	1.9 (0.9–3.9)			
**Tobacco**					Nil	
No	282	33 (11.7)	1	0.94		
Yes	79	9 (11.4)	1 (0.4–2.1)			

Number in dataframe = 361, Number in model = 361, Missing = 0, AIC = 197.5, C-statistic = 0.82, H&L = Chi-sq(8) 4.96 (P = 0.762)

### b Tinea versicolor

There were 11.4% of the participants infected with tinea versicolor. The prevalence in resettled villages (Village F, G, and H) was at 23.6% which was much higher than in inland villages (11.6%). Similarly, Bateq (15.5%) had a higher prevalence than non-Bateq participants (6.5%). Tinea versicolor was particularly pronounced in Village G (47.06%), a resettled Bateq villages. Furthermore, males displayed a higher prevalence (16.2%) compared to females (5.5%), and the overweight and obese group (24.1%) had a higher prevalence than underweight (9.0%) and normal (5.0%) groups (Table 4). Interestingly, 16.8% of the participants had family member(s) in the same household with tinea versicolor, which is more than double those who did not have any (7.0%). Pet owners (9.2%) had a lower prevalence than non-pet owners (14.2%).

In the univariate analysis (Table 4), Bateq subtribe (COR = 2.6, P = 0.009), males (COR = 3.3, P = 0.002), obese and overweight individuals (COR = 6.0, P = 0.001), presence of family members with tinea versicolor (COR = 2.7, P = 0.005), living in resettled villages (COR = 6.3, P < 0.001), and smokers (COR = 2.0, P = 0.047) had higher risks of getting tinea versicolor. Multivariate analysis showed Bateq subtribes (AOR = 4.4, P = 0.001), resettled villagers (AOR = 8.9, P < 0.001), males (AOR = 2.6, P = 0.040), obese and overweight individuals (AOR = 4.3, P = 0.015) and presence of family members with tinea versicolor (AOR = 3.5, P = 0.003) had a higher risk of getting tinea versicolor.

**Table 4:** Potential risk factors associated with tinea versicolor based on logistic regression analysis.

Variable	N	Tinea versicolor infected, n (%)	Univariate		Multivariate	
			COR (CI)	P value	AOR (CI)	P value
**Jahai subtribe**					Nil	
No	325	35 (10.8)	1	0.294		
Yes	36	6 (16.7)	1.7 (0.6–4.0)			
**Bateq subtribe**						
No	168	11 (6.5)	1	0.009*	1	0.001*
Yes	193	30 (15.5)	2.6 (1.3–5.7)		4.4 (1.9–10.9)	
**Gender**						
female	164	9 (5.5)	1	0.002*	1	0.040*
male	197	32 (16.2)	3.3 (1.6–7.6)		2.6 (1.1–6.7)	
**Body Mass Index**						
Normal	100	5 (5.0)	1		1	
Obese & overweight	83	20 (24.1)	6.0 (2.3–18.9)	0.001*	4.3 (1.4–15.1)	0.015*
Underweight	178	16 (9.0)	1.9 (0.7–5.9)	0.234	2.9 (0.9–10.4)	0.081
**Age group**					Nil	
Adult	210	28 (13.3)	1	0.166		
Kids and teens	151	13 (8.6)	0.6 (0.3–1.2)			
**Presence of pets**					Nil	
No	144	21 (14.6)	1	0.118		
Yes	217	20 (9.2)	0.6 (0.3–1.1)			
**Presence of family member with the same disease**						
No	200	14 (7.0)	1	0.005*	1	0.003*
Yes	161	27 (16.8)	2.7 (1.4–5.4)		3.5 (1.6–8.2)	
**Usage of topical ointments**					Nil	
No	244	32 (13.1)	1	0.133		
Yes	117	9 (7.7)	0.6 (0.2–1.2)			
**Occupations**					Nil	
Away from village	148	18 (12.2)	1	0.688		
Within village	213	23 (10.8)	0.9 (0.4–1.7)			
**Village status**						
Inland village	234	11 (4.7)	1	<0.001*	1	<0.001*
Resettled village	127	30 (23.6)	6.3 (3.1–13.6)		8.9 (3.7–23.8)	
**Tobacco**						
No	282	27 (9.6)	1	0.047*	1	0.23
Yes	79	14 (17.7)	2.0 (1.0–4.1)		1.7 (0.7–3.8)	

Number in dataframe = 361, Number in model = 361, Missing = 0, AIC = 203.9, C-statistic = 0.834, H&L = Chi-sq(8) 11.12 (P = 0.195)

### c Tinea imbricata

There were 7.5% participants who had tinea imbricate. More than half of those infected belonged to the inland Bateq subtribe in Villages C and D, underweight and not receiving any formal education. Chronic infections seemed to be a common trend, as 74.0% of them were infected with tinea imbricata for >100 weeks (~2 years) (S5 Table). In contrast with scabies and tinea versicolor, tinea imbricate had higher prevalence in inland villages (9.4%) compared to resettled villages (3.9%). Furthermore, female (9.1%) had higher prevalence than male (6.1%) (Table 5).

Both univariate and multivariate analyses (Table 5) highlighted that individuals from the Bateq subtribe (COR = 4.2, P = 0.005, AOR = 3.7, P = 0.011, respectively) and smokers (COR = 3.8, P = 0.001, AOR = 3.3, P = 0.004, respectively) had higher odds of getting tinea imbricata.

**Table 5:** Potential risk factors associated with tinea imbricata based on logistic regression analysis.

Variable	N	Tinea imbricata infected, n (%)	Univariate		Multivariate	
			COR (CI)	P value	AOR (CI)	P value
**Bateq subtribe**						
No	325	5 (3.0)	1	**0.005** *	1	**0.011** *
Yes	36	22 (11.4)	4.2 (1.7–12.8)		3.7 (1.4–11.3)	
**Gender**					Nil	
female	164	15 (9.1)	1	0.275		
male	197	12 (6.1)	0.6 (0.3–1.4)			
**Underweight**					Nil	
No	183	9 (4.9)	1	0.066		
Yes	178	18 (10.1)	2.2 (1.0–5.2)			
**Income level**					Nil	
<RM800	325	23 (7.1)	1	0.387		
>RM800	36	4 (11.1)	1.6 (0.5–4.6)			
**Age group**					Nil	
Adult	210	19 (9.0)	1	0.187		
Kids and teens	151	8 (5.3)	0.6 (0.2–1.3)			
**Presence of family member with the same disease**					Nil	
No	200	10 (5.0)	1	0.051		
Yes	161	17 (10.6)	2.2 (1.0–5.2)			
**Usage of topical ointments**					Nil	
No	244	23 (9.4)	1	0.052		
Yes	117	4 (3.4)	0.3 (0.1–0.9)			
**Occupations**					Nil	
Away from village	148	15 (10.1)	1	0.115		
Within village	213	12 (5.6)	0.5 (0.2–1.2)			
**Village status**					Nil	
Inland village	234	22 (9.4)	1	0.068		
Resettled village	127	5 (3.9)	0.4 (0.1–1.0)			
**Tobacco**						
No	282	14 (5.0)	1	**0.001** *	1	**0.004** *
Yes	79	13 (16.5)	3.8 (1.7–8.5)		3.3 (1.4–7.4)	

Number in dataframe = 361, Number in model = 361, Missing = 0, AIC = 180.2, C-statistic = 0.713, H&L = Chi-sq(8) 2.10 (P = 0.978)

## Discussion

Negrito communities are unique due to their distinct lifestyles, which differ from other indigenous tribes. These communities exhibit a broad spectrum of living conditions, ranging from isolated and primitive inland villages to resettled peripheral urban areas [30]. This diversity makes them well-suited for studying the interplay between urbanization and traditional lifestyles with regards to the prevalence of skin infections. There is a growing need for additional data to accurately assess the burden and impact of skin infections among the Negrito Orang Asli.

The present results revealed that skin infections remain a significant health concern within the Negrito communities in Peninsular Malaysia. More than 35.0% of the overall participants were found to have skin infections, with prevalence exceeding 50.0% in inland villages C, D, F and G. Despite belonging to the same Negrito tribe, villages A, B, E and H have significantly lower prevalence of skin infections compared to the other villages. The low prevalence of village A and B were perhaps associated with high frequency of medical interventions, which were effective in regulating the skin infections in those areas [31]. There was also no co-infection between scabies and tinea found in our study, which is consistent with other epidemiological studies showing that scabies is associated with pyoderma [32] and impetigo [14], rather than tinea. Co-infections of scabies and tinea appear to be associated to severely immunocompromised individuals [33].

It is interesting to note that the prevalence of skin infections in resettled villages was higher (55.2%) compared to inland villages (24.8%). Similar trend was observed when it comes to other tropical diseases such as helminthiasis [34]. Although it is generally hypothesized that better economic and infrastructure provision are negatively correlated with prevalence of infections as resettled villages typically result in provision of amenities, better accessibilities, infrastructure compared to the inland villages as per outlined in Lin et al., 1994 [35], the current findings suggest that several factors may counter this trend. Resettlement often involves merging of multiple Orang Asli communities into a single, larger village, leading to higher population density [35] which can facilitate the transmission of skin diseases, especially scabies. Moreover, there are varying levels of development in the resettlement scheme depending on the economic development of the country, regular provision by the authorities, and compliance by the villages [35]. Exposure to semi-urbanization or urbanization has also caused the younger generation of Negrito Orang Asli to be uninterested in agriculture, their traditional activity due to exposure to urban lifestyles. At the same time, some may also not be interested to work in the semi-urban or urban areas as they are unable to adapt to urban lifestyles due to inadequate education attainment [36], which lead to urban poverty issues and inability to pay for water and electricity bills or afford nutritious diets although there is provision of these basic amenities.

Furthermore, there seems be an association between subtribe and disease predisposition, suggesting the complex interplay of lifestyle and living habits influence on the likelihood of skin infections. For example, Kensiu subtribe displayed strong predisposition toward scabies as all of them resided in the village F (the sole Kensiu village in Malaysia). The Kensiu people live in the resettled village and they are more prone to higher inflation rates due to closer proximity to the urban areas resulting in urban poverty [37,38]. This was further supported by news coverage from 2020 indicating that the villagers could not sustain their livelihood in their resettled area and were forced to return to the inland forest for sustenance [39]. Moreover, unlike those in inland areas, these villagers could not rely on forest resources as part of their diet and for non-regular side income (eco-tourism, forest resource scavenging) [35]. This phenomenon leads to the inability to purchase sufficient foods, resulting in malnutrition [40] (40.0% of the participants were underweight and 32.5% were overweigh/obese, highlighting the phenomenon of double-burden malnutrition [41]). In addition, the Kensiu village is densely populated areas with abundant vegetation and shade, contributing to reduced sunlight exposure. It has been reported that factors which may facilitate the transmission of mites include increased contact among villagers and limited sunlight exposure [42].

On the other hand, different Bateq subtribes displayed different infection patterns. The Bateq in the inland villages, Village C (42.11%) and Village D (33.33%) had high prevalence of tinea imbricata whereas Bateq in resettled Village G (47.06%) displayed high prevalence of tinea versicolor. The higher prevalence of tinea imbricata among the inland villages, could be attributed to the smaller population size and gene pool, which might lead to the higher prevalence of susceptible genes due to inbreeding [43] as well as the higher exposure to the spores which remains in the areas [44]. However, the low prevalence of tinea imbricata in inland villages A and B was attributed to media coverage of tinea outbreaks in 2018 and 2019 [23,25] which prompted active intervention by health authorities and the non-governmental organizations (NGOs). Health care officers visited these villages frequently, providing villagers with medications and consistent monitoring to ensure good treatment compliance and follow-up [45,46]. It is important to note that, tinea imbricata is curable with anti-fungal such as terbinafine [47]. The key to controlling these infections lies in follow-up to ensure patient compliance. Medical intervention and follow-up are effective measures in controlling this infection [31].

**Fig 1:**
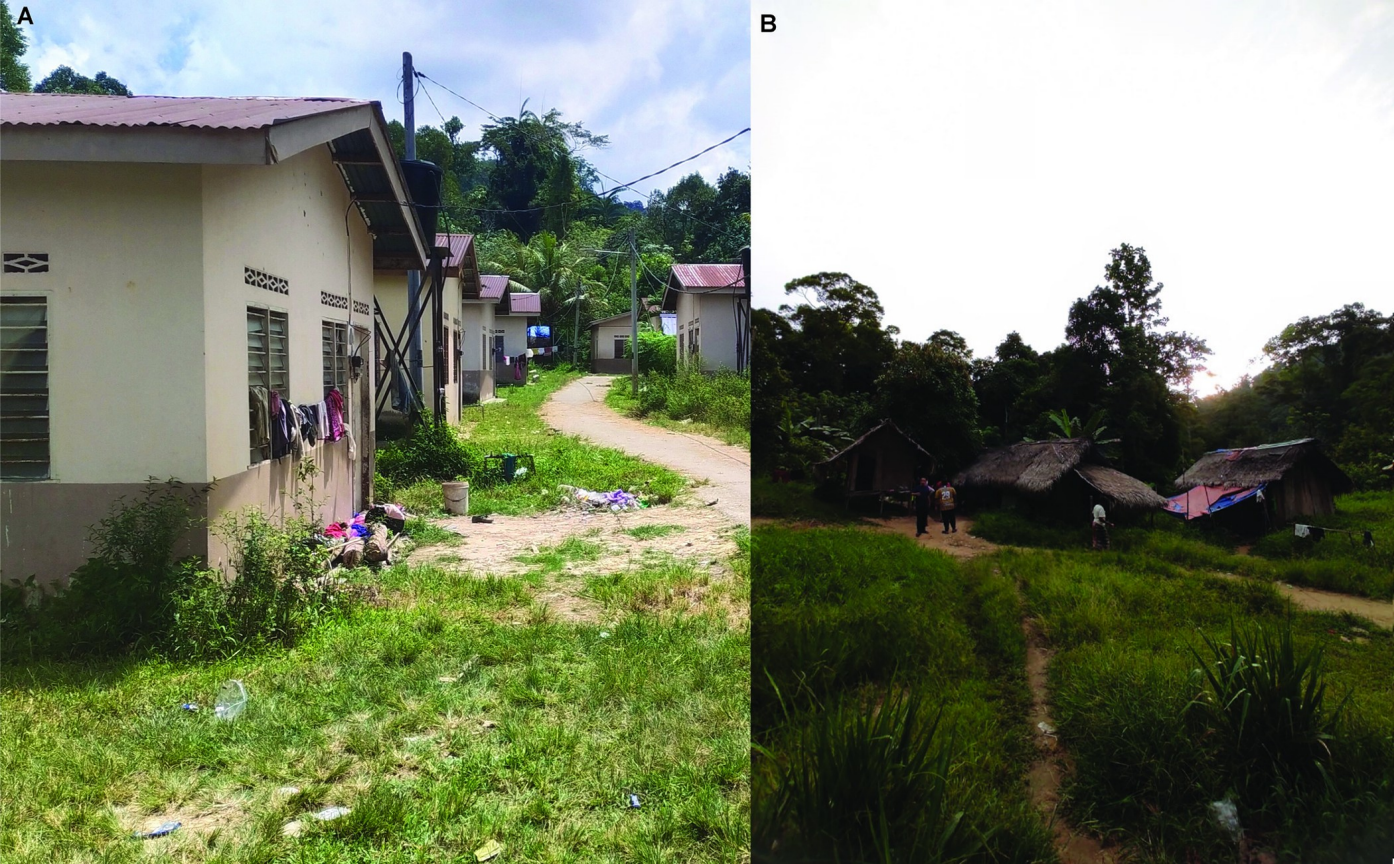
Village architecture of Bateq people. **(A)** Bateq inland village which has limited tall vegetation and shaded areas leading to high sunlight exposure. **(B)** In this resettled village, villagers stay in government-built brick houses.

On the other hand, the high prevalence of tinea versicolor among resettled Bateq village could be attributed to the village surrounding environment and housing design (Fig 1A and 1B). In this village, there was less vegetation, resulting in limited shaded areas and high exposure to sunlight and higher temperature [48]. The high temperature which promote perspiration could induce higher risk of tinea versicolor [49], particularly in the resettled villages where the villagers were staying in brick houses (Fig 1B) which lack ventilation [50].

Scabies was the most common skin infection (11.6%) among the Negritos in this study and there have been no prior publications about its prevalence among this community. However, the prevalence found in this study is similar to that reported among the Temiar Orang Asli communities in 1998 in Pos Piah, Perak (11.9%) [17] and in suburban communities in Jengka Triangle, Pahang (11.6%) [51]. Comparatively, its prevalence is only slightly higher compared to the prevalence reported in an urban population survey in Kuching, East Malaysia (8.1%) [52] indicating that scabies are not limited to lower-income groups or less-developed areas. Further analysis indicated scabies was most prevalent (60.0%) in village F, supporting the latter statement despite the village’s closer proximity to urban areas and access to basic infrastructure. Most of the cases were found among individuals categorized as underweight (14.6%), which correlate with the global research, that malnourish [53] was the key risk factors of scabies.

On the other hand, the prevalence of tinea versicolor was 11.4%, primarily observed in villages D and G, which is higher than the earlier study among the Negrito communities of Perak in 1952 (6.1%) [4] and among Senoi tribe in Pos Piah (1.7%) [17]. It is interesting to note that the prevalence of the current study is lower than the finding among the urban population in Singapore (25.2%) [54]. Globally, poverty [55], hygiene practices and sanitary infrastructure [56], treatment compliance [57] and malnutrition [58] and genetic [43] have been associated with the prevalence of tinea. Similar to the study in Brazil and China [59,60], males had higher odds of getting tinea versicolor. This is likely due to males having higher testosterone levels, which results in more active sweat glands [61], which promote the pathogenesis of tinea versicolor [58]. Moreover, obese and overweight group had higher odds of getting tinea versicolor due to a lower tolerance of heat and hence lead to more active sweat glands and perspiration rates [62]. Furthermore, the presence of a family member with the same infection was identified as a risk factor for tinea versicolor. This can be attributed to to complex polyclonal inheritance nature of tinea versicolor [59], meaning there are multiple genetic factors involved. The presence of a family member with tinea versicolor indicates the presence of susceptibility gene sets, which increases the likelihood of developing the condition.

Finally, the prevalence of tinea imbricata in our study (7.5%) was much lower than the report in 1952 among the Negrito communities in Perak (18.3%) [4]. The present prevalence data among Negrito is also lower compared to the observations among the Semai tribe (12.5%) in 1952, but higher compared to the Proto-Malay tribe (4.4% in 1952) [4] and among urban population (3.5% in 2002) [63]. Our multivariate binomial logistic regression model showed that smoking or the use of tobacco is an important risk factor for tinea imbricata. In this context, smoking leads to immunomodulation of the host’s immunity through the generation of platelet-activating factor agonists [64] and physical changes to the skin, including lowering epidermis and dermis thickness [65]. These factors may increase the susceptibility of smokers to tinea infections compared to non-smokers.

### Limitation and future studies

Cross-sectional study such as this has inherent limitations particularly in its ability to provide only a snapshot in time, limiting the capture of dynamic changes in disease patterns and hindering the establishment of causal relationships. A longitudinal study would be essential to obtain a more comprehensive understanding of the temporal dynamics of skin infections within the indigenous population. Moreover, there is variation of the village sizes, especially the inland or nomadic to semi-nomadic villages are much smaller than those resettled villages due to mobility and logistic issues, therefore posed significant challenges in standardizing sample sizes. Future research should focus on intrinsic and biotic factors including the microbiome, hormones, cytokines, and host genetics to gain comprehensive insights into the causes and persistence of skin infections in the Negrito communities, enabling the development of tailored preventive and therapeutic strategies.

## Conclusion

This study highlighted the current burden of skin infections among the Negritos revealing a higher prevalence than previously reported. Our results displayed a strong predisposition of different infections toward different village settings: scabies and tinea versicolor are more prevalent in resettled and more advanced villages, whereas tinea imbricata is primarily found in the inland villages. Other than that, villages with the same subtribe had different predispositions due to variation in the level of development and frequency of medical interventions. Other contributing factors include lifestyle practices, such as smoking, can weaken host immunity and elevate the risk of skin infections. Variations in village lifestyles, housing conditions, population density, and sunlight exposure also influence disease risk. To combat these infections effectively, tailored approaches are needed for different village types. Inland villages may benefit from scheduled medical interventions, while resettled communities could require socio-economic support to alleviate urban or semi-urban poverty. These strategies acknowledge the varying susceptibility to skin infections observed across different village settings.

## Material and methods

### a Ethics application

This study was approved by the Medical Ethics Committee of the University of Malaya Medical Centre (UMMC) (Reference No.: 20201030–9172) to collect skin samples, photos, and questionnaires from participants and permission was also obtained from the Department of Orang Asli Development (JAKOA) [Reference No.: JAKOA.PP.30.032 JLD 52 (34)]. Additional consultations were obtained from the JAKOA official at the district level and villages’ chieftains (referred to as ‘Tok Batin’) prior to commencing the research in the respective areas and villages. The purpose and procedures of the study were verbally explained to the villagers and printed form (both in Bahasa Melayu and English) were also provided before the commencement of the research. The interview and consent approval were conducted in Bahasa Melayu, the national language of Malaysia. If participants were unable to comprehend Bahasa Melayu, a village interpreter will be involved in translating. Prior to inclusion in this study, written consent was obtained from all participants. For children under 18 years old, an assent was obtained followed by written consent from their parents or guardians.

### b Study area and study population

**Fig 2:**
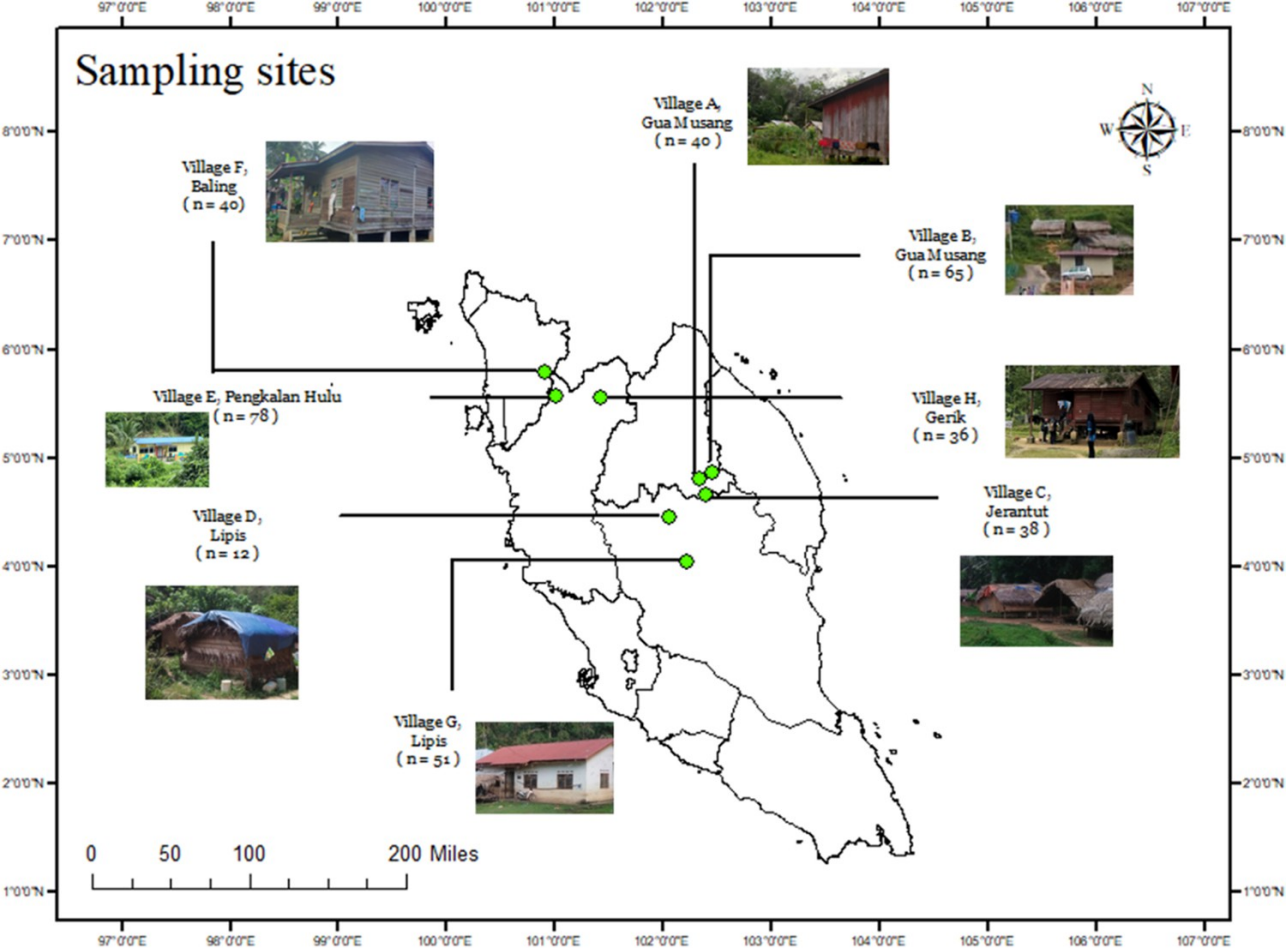
Location of studies sites. Different villages showed different levels of development. (plotted with ArcMap v10.7, sources: Esri, Michael Bauer Research GMBH 2022, Department of Statistics Malaysia [67].

This cross-sectional study was conducted between November 2021 and March 2023 in eight Negrito villages located in the Northern and Central Peninsular Malaysia (Fig 2). The focus on Negrito tribe was based on the study by Polunin in 1952, which indicated that Negrito tribe was the most prone to skin infections. The approval to conduct study at the villages was based on the discussions with the central and district/local JAKOA officers. Additionally, the opinions and receptiveness of the village committee and village chieftain were also taken into consideration. The villages identified were of different levels of economic activity, infrastructure availability, and accessibility to assess the impact of development on the prevalence of skin infections (Table 6). Native tongue was the primary language of the villagers though they had a good, or at least satisfactory, command of the Bahasa Melayu.

**Table 6:** Characteristics of each village.

Village	Subtribe	Number of participants	Water sources	Accessibility	Distance from town/ hr	Main economic	Village status
A	Bateq	40	River	Tar road	2	Agriculture	Inland
B	Bateq	66	River	Tar road and mud road	2	Forestry	Inland
C	Bateq	38	River	Boat only	2.5	Forestry	Inland
D	Bateq	12	River	Tar road	2.5	Forestry	Inland
E	Kintak	78	River	Tar road	1	Agriculture	Inland
F	Kensiu	40	River and treated water	Tar road	0.16	Industry, tourism, Agriculture	Resettled
G	Bateq	51	River and treated water	Tar road	0.6	Agriculture	Resettled
H	Jahai	36	River	Tar road	0.6	Agriculture	Ressetled

*Resettled village refer to those where the entire villages was moved under the Resttlement plan scheme (RPS) (34)

There are 2 main categories of villages, the resettled village that have been relocated under the government’s resettlement plan scheme, with the new location near the township, with housing, water and electricity infrastructure provided [66] and those which maintained their traditional lifestyle which may include being semi-nomadic in the inland/remote villages. The resettled villages are those villages which participated in the relocation scheme collectively called *as Rancangan Pengumpulan Semula* (RPS) since 1970s whereas inland village refer to those villages which are still situated in their original locations. In brief, resettled villages were relocated by the Department of Orang Asli Development (JAKOA) from interior to a more accessible location together with promises of multiple development scheme including basic infrastructure and facility, economic subsidy, economic diversification programme (eg., ALDP [Agriculture Land Development Programme]) and eco-tourism [35].

### c Sampling location

Sampling sites were determined through multi-stakeholder discussions as described previously. Upon institutional IRB approval, discussion sessions were held with the JAKOA officers to explain the rationale and aims of the study. The JAKOA officers determined the suitability of the villages, for example, whether the village chieftain, committee and villagers are receptive to the research activities, accessibility and remoteness of the villages (certain villages can only be reached by helicopter), fluency of national language as the research is conducted in the national language.

Prior to the study being conducted in the villages, JAKOA officers accompanied researchers to the villages for meetings with the Tok Batin (the chieftain of the village) to further explain the rationale and aims of the study. Following that, the whole village was invited for a briefing and dialogue session and samples were only taken from individuals who gave consent after understanding the nature of the research.

Therefore, engagement prior to sampling was according to the national rules and regulations of research conduct in these minority communities, adhering and respecting sensitive cultural beliefs and practices.

### d Sample collection

Power calculation developed by Cohen, 1988 [68] were employed to calculate our sample sizes via R package PWR [69]. By including desired power of 90% and significant level to 0.01, the script would be as the next paragraph, with the minimum sample sizes of 231 for scabies study and 156 for tinea study (Table 7).

pwr.2p.test(h = ES.h(p1 = prevalence rate 1, p2 = prevalence rate 2), sig.level = 0.01, power = .90,alternative = "greater")

**Table 7:** Reference and output for sample size calculation.

Disease	Prevalence (%)	Number of sample (n)	Sample sizes/n	Effect sizes/h
Scabies	24.7% (21)	312	**231**	0.34
	11.9% (17)	356		
Tinea	17.4% (4)	581	**156**	0.41
	5% (17)	356		

Individuals with a minimum age of 3 years old, who had resided in the village for at least a year were included in our study. The children below 3 years old were excluded from our study due to potential challenges in obtaining their assent and also the concerns about the use of glass slides during sample collection. Given that children below 3 years old might find the process distressing, it could negatively impact the participation rates in the study. Besides, non-Negrito individuals who resided in the village or Negrito individuals who were only staying in the village temporarily were also excluded from the study.

**Fig 3:**
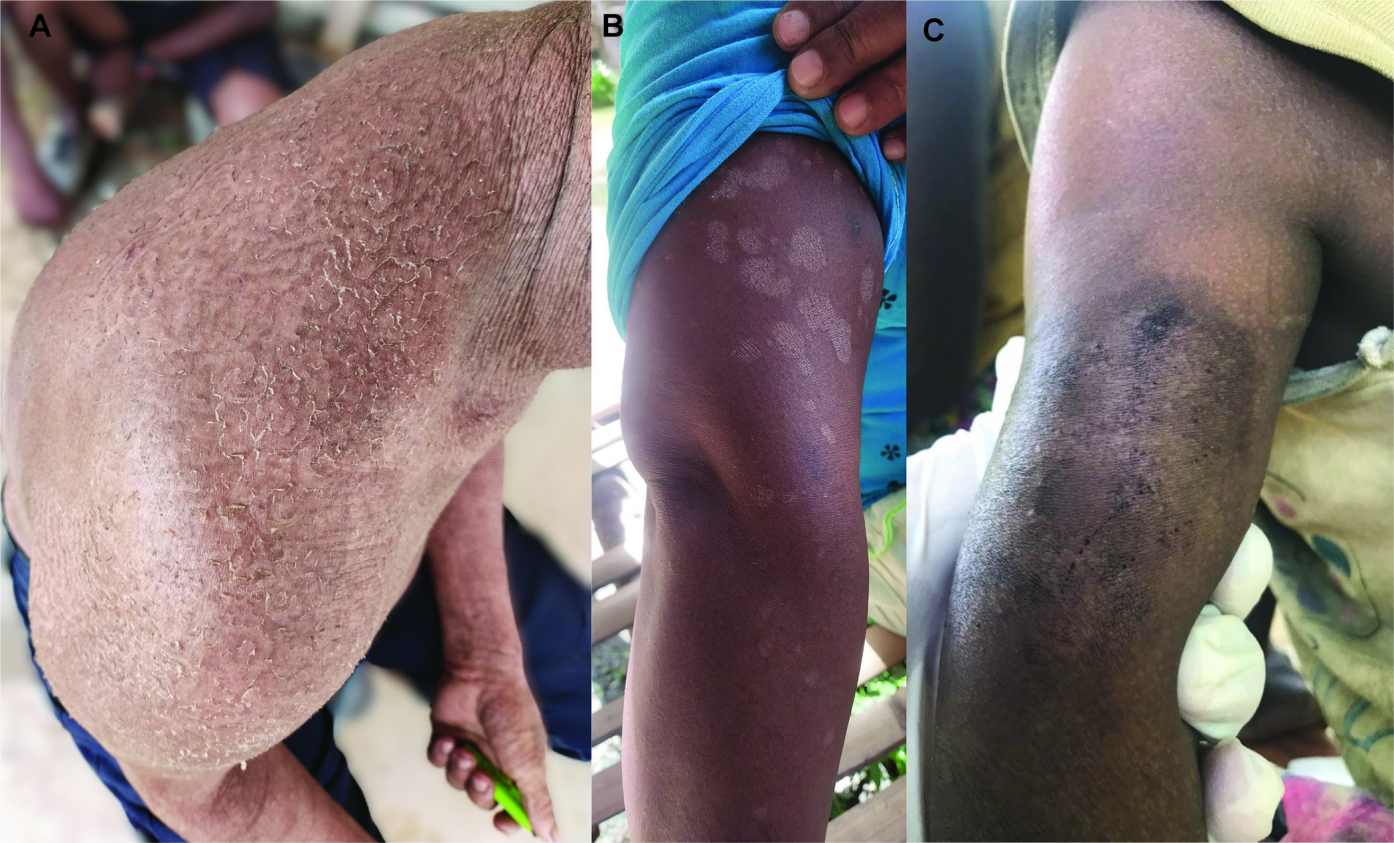
Photo of skin infections. **(A)** tinea imbricata (**B)** tinea versicolor and (**C)** scabies (Photo taken by Yi Xian, Er after getting the consent from the participants).

Skin scraping samples were collected from the participants following the description by Ponka (2014) [70]. Brand-new glass slides were used to gently scrape the skin and the samples were collected onto black sugar paper. The samples were then placed in pre-labelled envelopes with the participant’s name and identity code. All the skin samples were preserved at ambient temperature and the microscopy and culture test were carried out on the same day. For participants with tinea lesions, we followed the CLSI-m54a methods described by the Clinical and Laboratory Standards Institute (CLSI). The skin scrapings were inoculated onto pre-labelled Mycosel agar with a sample ID and date [71]. The agar was incubated at room temperature, and photos were taken every week for a period of eight weeks. A photo of the lesion was taken upon obtaining consent from the participant (Fig 3).

A dual-language pre-tested interview-based questionnaire in English and Bahasa Melayu was conducted face-to-face in Bahasa Melayu. For young children, the interview was carried out with the assistance of their guardians. The information collected included basic demographic data, socioeconomic status, education level, lifestyle factors, source of water supply, infrastructure availability, personal hygiene practices, as well as history of medication and skin infections. Height and weight were measured and recorded as well. The BMI was calculated and categorized into four categories (obese, overweight, normal and underweight) based on the standard set up by WHO [72] and the Ministry of Health, Malaysia.

### c Detection of scabies and tinea

**Fig 4:**
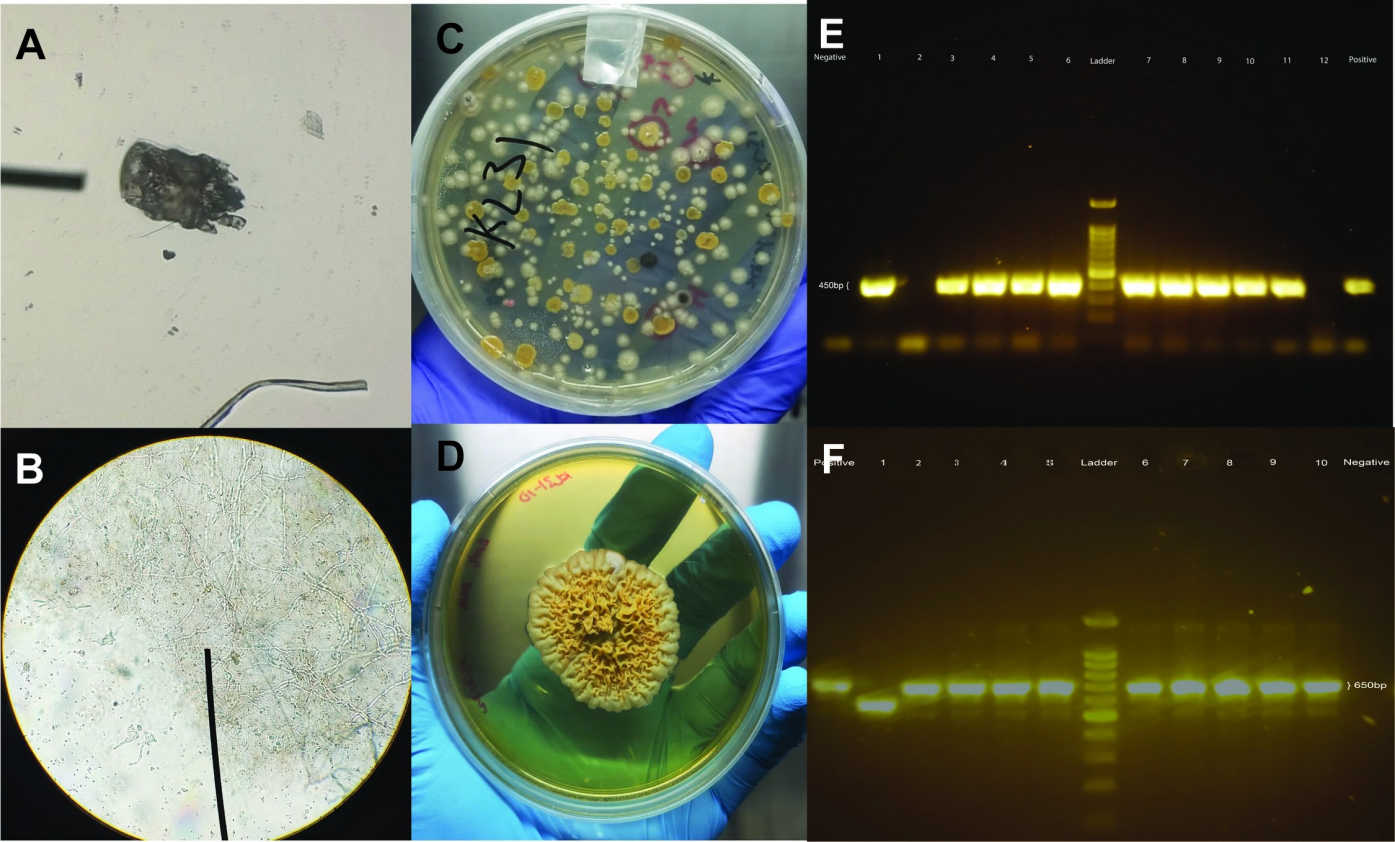
Photo of skin infections via microscopy and PCR. (**A)** Mites in KOH processed skin sample (**B)** Hyphal elements in KOH processed skin sample; (**C)** Mycosel plates showing fungal colonies with red arrows pointing at presumptive colonies of dermatophytes; (**D)** *T*. *concentricum* colony subcultured from the primary Mycosel plate; (**E)** Agarose gel electrophoresis of the Pan-derm PCR products (~450bp); (**F)** Agarose gel electrophoresis of the ITS1-4 PCR products (~650bp).

The potassium hydroxide (KOH) skin test was used for rapid detection of skin infections [70]. Briefly, the collected skin scrapings were inoculated onto the surface of a clean glass slide, followed by the addition of a drop of 20% KOH solution. The slide was then incubated at 60°C for 15 minutes. The samples were covered with cover slips and observed under light microscope. The presence of hyphal elements or mites was recorded in writing and/or by taking photographs (see Fig 4A and 4B).

For the collected Mycosel agar (Fig 4C), unique fungal colonies were sub-cultured onto Sabouraud dextrose agar (Fig 4D). The DNA was then extracted using the heat treatment method [73]. Polymerase chain reaction (PCR) (panDerm-PCR and ITS PCR) was used for the rapid confirmation of dermatophytes, utilizing the dermatophyte-specific chitin synthase primer (panDerm) [74] (Fig 4E). The isolates that tested positive were further subjected to Internal Transcribed Spacer PCR to confirm their identities [75] (Fig 4F).

### d Statistical analysis

The data entry of the collected questionnaire was done using Microsoft Excel Pro 2019. The output file was saved in the form of comma-separated files (CSV) and stored for analysis. Statistical analysis was performed using R-4.1.2. The display graphs were created using R Statistical Software (version 4.0.3).

The discrete variables and demographic data of the study population (demographic, socioeconomic, lifestyles, and personal hygiene practices) and incidence rates of skin infections were expressed as percentages and visualized using either the ggplot2 or ggpubr package [76,77]. The normality of all continuous variables was evaluated using the Shapiro-Wilk test, with normality defined as P > 0.05.

For the risk factor analysis, Pearson’s chi-square test was used to determine the association between skin infections (dependent variable) with various lifestyle and demographic variables. A cross-table output was generated using the R finalfit package [78]. The significant variables were then included in a binomial logistic regression analysis, which attempts to predict the probability that an observation falls into one of two categories of a dichotomous dependent variable based on one or more independent variables (either continuous or categorical, using the finalfit package of R [78]. The output data was interpreted using adjusted odds ratios and 95% confidence intervals. The level of significance was set at P < 0.05.

## Supporting information

S1 Table Three main tribes and 18 subtribes of Orang Asli in Peninsular Malaysia. (DOCX)

S2 Table Cross-table analysis (Chi-square test) of scabies. (DOCX)

S3 Table Output of cross-table (Chi-square test) analysis of tinea versicolor. (DOCX)

S4 Table Output of cross-table (Chi-square test) analysis of tinea imbricata. (DOCX)

S5 Table Descriptive data of the tinea imbricata patients. (DOCX)
