# Supplementary material for: Infection patterns of scabies and tinea between inland and resettled indigenous Negrito communities in Peninsular Malaysia
Source: PLoS Negl Trop Dis. 2024 Sep 26;18(9):e0012515. doi: 10.1371/journal.pntd.0012515 (PMC11460705; doi:10.1371/journal.pntd.0012515)
Supplement: S1 Table — (DOCX) [file pntd.0012515.s001.docx]

Supplementary Table 1: Three main tribes and 18 subtribes of Orang Asli in Peninsular Malaysia

| Tribes | Senoi (N= 118,138) | Negrito (N= 6,546) | Proto-Malay (N= 90,531) |
| --- | --- | --- | --- |
| Sub-tribes | - Semai - Temiar - Jah Hut - Semaq Beri - Mah meri - Cheq Wong | - Mandriq - Bateq - Jahai - Kensiu - Kintaq - Lanoh | - Jakun - Semelai - Temuan - Orang Seletar - Orang Kuala - Orang Kanaq |
