# Supplementary material for: Infection patterns of scabies and tinea between inland and resettled indigenous Negrito communities in Peninsular Malaysia
Source: PLoS Negl Trop Dis. 2024 Sep 26;18(9):e0012515. doi: 10.1371/journal.pntd.0012515 (PMC11460705; doi:10.1371/journal.pntd.0012515)
Supplement: S2 Table — (DOCX) [file pntd.0012515.s002.docx]

Supplementary Table 2: Cross-table analysis (Chi-square test) of scabies

| **Variables** | **Entity** | **Number/Percentage of those** | | **p-value** |
| --- | --- | --- | --- | --- |
|  |  | **No** | **Yes** |  |
| Village | Village A | 37 (11.6) | 3 (7.1) | <0.001 |
|  | Village H | 36 (11.3) | 0 (0.0) |  |
|  | Village E | 71 (22.3) | 7 (16.7) |  |
|  | Village D | 12 (3.8) | 0 (0.0) |  |
|  | Village B | 64 (20.1) | 2 (4.8) |  |
|  | Village F | 16 (5.0) | 24 (57.1) |  |
|  | Village C | 35 (11.0) | 3 (7.1) |  |
|  | Village G | 48 (15.0) | 3 (7.1) |  |
| Subtribe | Bateq | 182 (57.1) | 8 (19.0) | <0.001 |
|  | Jahai | 36 (11.3) | 0 (0.0) |  |
|  | Kensiu | 16 (5.0) | 19 (45.2) |  |
|  | Kintak | 71 (22.3) | 7 (16.7) |  |
|  | Mandriq and Lanoh | 14 (4.4) | 8 (19.0) |  |
| Gender | Female | 145 (45.5) | 19 (45.2) | 1 |
|  | Male | 174 (54.5) | 23 (54.8) |  |
| Body Mass Index | Normal | 95 (29.8) | 5 (11.9) | 0.049 |
|  | Obese and overweight | 72 (22.6) | 11 (26.2) |  |
|  | Underweight | 152 (47.6) | 26 (61.9) |  |
| Age group | Adult | 188 (58.9) | 22 (52.4) | 0.52 |
|  | Kids and teen | 131 (41.1) | 20 (47.6) |  |
| Education | No formal education | 142 (44.5) | 21 (50.0) | 0.612 |
|  | With Education | 177 (55.5) | 21 (50.0) |  |
| Income | <800 | 298 (93.4) | 27 (64.3) | <0.001 |
|  | >800 | 21 (6.6) | 15 (35.7) |  |
| Water status | Mix | 55 (69.6) | 24 (30.4) | <0.001 |
|  | Treated | 36 (85.7) | 6 (14.3) |  |
|  | Untreated | 228 (95.0) | 12 (5.0) |  |
| Presence of pets | No | 129 (40.4) | 15 (35.7) | 0.674 |
|  | Yes | 190 (59.6) | 27 (64.3) |  |
| Presence of family member with the same infection | No | 174 (54.5) | 26 (61.9) | 0.461 |
|  | Yes | 145 (45.5) | 16 (38.1) |  |
| Usage of topical ointments | No | 226 (70.8) | 18 (42.9) | 0.001 |
|  | Yes | 93 (29.2) | 24 (57.1) |  |
| Tobacco | No | 249 (78.1) | 33 (78.6) | 1 |
|  | Yes | 70 (21.9) | 9 (21.4) |  |
| Village status | Inland village | 219 (68.7) | 15 (35.7) | <0.001 |
|  | Resettled village | 100 (31.3) | 27 (64.3) |  |
| Occupations | Away from village | 136 (42.6) | 12 (28.6) | 0.115 |
|  | Within village | 183 (57.4) | 30 (71.4) |  |
