# Supplementary material for: Infection patterns of scabies and tinea between inland and resettled indigenous Negrito communities in Peninsular Malaysia
Source: PLoS Negl Trop Dis. 2024 Sep 26;18(9):e0012515. doi: 10.1371/journal.pntd.0012515 (PMC11460705; doi:10.1371/journal.pntd.0012515)
Supplement: S3 Table — (DOCX) [file pntd.0012515.s003.docx]

Supplementary Table 3: Output of cross-table (Chi-square test) analysis of tinea versicolor

| **Variables** | **Entity** | **Number/Percentage of those** | | **p-value** |
| --- | --- | --- | --- | --- |
|  |  | **No** | **Yes** |  |
| Village | Village A | 36 (11.2) | 4 (9.8) | <0.001 |
|  | Village H | 30 (9.4) | 6 (14.6) |  |
|  | Village E | 78 (24.4) | 0 (0.0) |  |
|  | Village D | 7 (2.2) | 5 (12.2) |  |
|  | Village B | 64 (20.0) | 2 (4.9) |  |
|  | Village F | 40 (12.5) | 0 (0.0) |  |
|  | Village C | 38 (11.9) | 0 (0.0) |  |
|  | Village G | 27 (8.4) | 24 (58.5) |  |
| Subtribe | Bateq | 160 (50.0) | 30 (73.2) | <0.001 |
|  | Jahai | 30 (9.4) | 6 (14.6) |  |
|  | Kensiu | 35 (10.9) | 0 (0.0) |  |
|  | Kintak | 78 (24.4) | 0 (0.0) |  |
|  | Mandriq and Lanoh | 17 (5.3) | 5 (12.2) |  |
| Gender | Female | 155 (48.4) | 9 (22.0) | 0.002 |
|  | Male | 165 (51.6) | 32 (78.0) |  |
| Body Mass Index | Normal | 95 (29.7) | 5 (12.2) | <0.001 |
|  | Obese and overweight | 63 (19.7) | 20 (48.8) |  |
|  | Underweight | 162 (50.6) | 16 (39.0) |  |
| Age group | Adult | 182 (56.9) | 28 (68.3) | 0.22 |
|  | Kids and teen | 138 (43.1) | 13 (31.7) |  |
| Education | No formal education | 145 (45.3) | 18 (43.9) | 0.997 |
|  | With Education | 175 (54.7) | 23 (56.1) |  |
| Income | <800 | 284 (88.8) | 41 (100.0) | 0.047 |
|  | >800 | 36 (11.2) | 0 (0.0) |  |
| Water status | Mix | 72 (22.5) | 7 (17.1) | <0.001 |
|  | Treated | 20 (6.2) | 22 (53.7) |  |
|  | Untreated | 228 (71.2) | 12 (29.3) |  |
| Presence of pets | No | 123 (38.4) | 21 (51.2) | 0.16 |
|  | Yes | 197 (61.6) | 20 (48.8) |  |
| Presence of family member with the same infection | No | 186 (58.1) | 14 (34.1) | 0.006 |
|  | Yes | 134 (41.9) | 27 (65.9) |  |
| Usage of topical ointments | No | 212 (66.2) | 32 (78.0) | 0.179 |
|  | Yes | 108 (33.8) | 9 (22.0) |  |
| Tobacco | No | 255 (79.7) | 27 (65.9) | 0.069 |
|  | Yes | 65 (20.3) | 14 (34.1) |  |
| Village status | Inland village | 223 (69.7) | 11 (26.8) | <0.001 |
|  | Resettled village | 97 (30.3) | 30 (73.2) |  |
| Occupations | Away from village | 130 (40.6) | 18 (43.9) | 0.816 |
|  | Within village | 190 (59.4) | 23 (56.1) |  |
