# Supplementary material for: Infection patterns of scabies and tinea between inland and resettled indigenous Negrito communities in Peninsular Malaysia
Source: PLoS Negl Trop Dis. 2024 Sep 26;18(9):e0012515. doi: 10.1371/journal.pntd.0012515 (PMC11460705; doi:10.1371/journal.pntd.0012515)
Supplement: S4 Table — (DOCX) [file pntd.0012515.s004.docx]

Supplementary Table 4: Output of cross-table (Chi-square test) analysis of tinea imbricata

| **Variables** | **Entity** | **Number/Percentage of those** | | **p-value** |
| --- | --- | --- | --- | --- |
|  |  | **No** | **Yes** |  |
| Village | Village A | 40 (12.0) | 0 (0.0) | <0.001 |
|  | Village H | 36 (10.8) | 0 (0.0) |  |
|  | Village E | 78 (23.4) | 0 (0.0) |  |
|  | Village D | 8 (2.4) | 4 (14.8) |  |
|  | Village B | 64 (19.2) | 2 (7.4) |  |
|  | Village F | 35 (10.5) | 5 (18.5) |  |
|  | Village C | 22 (6.6) | 16 (59.3) |  |
|  | Village G | 51 (15.3) | 0 (0.0) |  |
| Subtribe | Bateq | 168 (50.3) | 22 (81.5) | 0.001 |
|  | Jahai | 36 (10.8) | 0 (0.0) |  |
|  | Kensiu | 30 (9.0) | 5 (18.5) |  |
|  | Kintak | 78 (23.4) | 0 (0.0) |  |
|  | Mandriq and Lanoh | 22 (6.6) | 0 (0.0) |  |
| Gender | Female | 149 (44.6) | 15 (55.6) | 0.369 |
|  | Male | 185 (55.4) | 12 (44.4) |  |
| Body Mass Index | Normal | 91 (27.2) | 9 (33.3) | 0.012 |
|  | Obese and overweight | 83 (24.9) | 0 (0.0) |  |
|  | Underweight | 160 (47.9) | 18 (66.7) |  |
| Age group | Adult | 191 (57.2) | 19 (70.4) | 0.257 |
|  | Kids and teen | 143 (42.8) | 8 (29.6) |  |
| Education | No formal education | 147 (44.0) | 16 (59.3) | 0.183 |
|  | With Education | 187 (56.0) | 11 (40.7) |  |
| Income | <800 | 302 (90.4) | 23 (85.2) | 0.59 |
|  | >800 | 32 (9.6) | 4 (14.8) |  |
| Water status | Mix | 65 (19.5) | 14 (51.9) | <0.001 |
|  | Treated | 40 (12.0) | 2 (7.4) |  |
|  | Untreated | 229 (68.6) | 11 (40.7) |  |
| Presence of pets | No | 133 (39.8) | 11 (40.7) | 1 |
|  | Yes | 201 (60.2) | 16 (59.3) |  |
| Presence of family member with the same infection | No | 190 (56.9) | 10 (37.0) | 0.073 |
|  | Yes | 144 (43.1) | 17 (63.0) |  |
| Usage of topical ointments | No | 221 (66.2) | 23 (85.2) | 0.069 |
|  | Yes | 113 (33.8) | 4 (14.8) |  |
| Tobacco | No | 268 (80.2) | 14 (51.9) | 0.001 |
|  | Yes | 66 (19.8) | 13 (48.1) |  |
| Village status | Inland village | 212 (63.5) | 22 (81.5) | 0.094 |
|  | Resettled village | 122 (36.5) | 5 (18.5) |  |
| Occupations | Away from village | 133 (39.8) | 15 (55.6) | 0.163 |
|  | Within village | 201 (60.2) | 12 (44.4) |  |
