# Supplementary material for: Infection patterns of scabies and tinea between inland and resettled indigenous Negrito communities in Peninsular Malaysia
Source: PLoS Negl Trop Dis. 2024 Sep 26;18(9):e0012515. doi: 10.1371/journal.pntd.0012515 (PMC11460705; doi:10.1371/journal.pntd.0012515)
Supplement: S5 Table — (DOCX) [file pntd.0012515.s005.docx]

Supplementary Table 5: Descriptive data of the tinea imbricata patients

| **Group** | **Variable** | **Number** | **Percentage/%** |
| --- | --- | --- | --- |
| Village | **Village C** | **16** | **74.07** |
|  | Village D | 3 | 33.33 |
|  | Village F | 5 | 18.52 |
|  | Village B | 2 | 7.41 |
| State | **Pahang** | **20** | **74.07** |
|  | Kedah | 5 | 18.52 |
|  | Kelanatan | 2 | 7.41 |
| Subtribe | **Bateq** | **22** | **81.48** |
|  | Kensiu | 5 | 18.52 |
| Gender | Male | 15 | 55.56 |
|  | Female | 12 | 44.44 |
| BMI | **Underweight** | **18** | **66.67** |
|  | Normal | 9 | 33.33 |
| Age group | Adult | 19 | 70.37 |
|  | Kids | 8 | 29.63 |
| Pruritus | No | 16 | 59.26 |
|  | Yes | 11 | 40.74 |
| Education | **No formal education** | **16** | **59.26** |
|  | Primary education | 6 | 22.22 |
|  | Secondary education | 5 | 18.52 |
| Income | **<800** | **25** | **92.59** |
|  | >800 | 2 | 7.41 |
| Meals /days | 2 | 17 | 62.96 |
|  | 3 | 10 | 37.04 |
| Usage of untreated water | **Yes** | **25** | **92.59** |
|  | No | 2 | 7.41 |
| Number of bath/days | 3 | 13 | 48.15 |
|  | 2 | 9 | 33.33 |
|  | >3 | 5 | 18.52 |
| Usage of soap | Always | 25 | 92.59 |
|  | Sometimes | 2 | 7.41 |
| Forest activity (Playing, working, hunting) | No | 15 | 55.56 |
|  | Yes | 12 | 44.44 |
| Cleaning after coming from outdoors | Sometimes | 13 | 48.15 |
|  | Always | 8 | 29.63 |
|  | Never | 6 | 22.22 |
| Pets’ availability | Yes | 16 | 59.26 |
|  | No | 11 | 40.74 |
| Close contacts with pets | No | 19 | 70.37 |
|  | Yes | 8 | 29.63 |
| Weeks of having tinea imbricate | **>100 weeks** | **20** | **74.07** |
|  | <100 weeks | 7 | 25.93 |
| Presence of family member with tinea imbricata | **Yes** | **17** | **62.96** |
|  | No | 10 | 37.04 |
| Smoking | No | 14 | 51.85 |
|  | Yes | 13 | 48.15 |
